# Measuring Technology-Facilitated Sexual Violence and Abuse in the Chinese Context: Development Study and Content Validity Analysis

**DOI:** 10.2196/65199

**Published:** 2024-11-19

**Authors:** Sharon Hoi Lam Pak, Chanchan Wu, Kitty Wai Ying Choi, Edmond Pui Hang Choi

**Affiliations:** 1 School of Nursing Li Ka Shing Faculty of Medicine The University of Hong Kong Hong Kong China (Hong Kong)

**Keywords:** technology-facilitated sexual violence and abuse, TFSVA, image-based sexual abuse, sexual abuse, content validity, measurement, questionnaire, China

## Abstract

**Background:**

Technology-facilitated sexual violence and abuse (TFSVA) encompasses a range of behaviors where digital technologies are used to enable both virtual and in-person sexual violence. Given that TFSVA is an emerging and continually evolving form of sexual abuse, it has been challenging to establish a universally accepted definition or to develop standardized measures for its assessment.

**Objective:**

This study aimed to address the significant gap in research on TFSVA within the Chinese context. Specifically, it sought to develop a TFSVA measurement tool with robust content validity, tailored for use in subsequent epidemiological studies within the Chinese context.

**Methods:**

The first step in developing the measurement approach for TFSVA victimization and perpetration was to conduct a thorough literature review of existing empirical research on TFSVA and relevant measurement tools. After the initial generation of items, all the items were reviewed by an expert panel to assess the face validity. The measurement items were further reviewed by potential research participants, who were recruited through snowball sampling via online platforms. The assessment results were quantified by computing the content validity index (CVI). The participants were asked to rate each scale item in terms of its relevance, appropriateness, and clarity regarding the topic.

**Results:**

The questionnaire was reviewed by 24 lay experts, with a mean age of 27.96 years. They represented different genders and sexual orientations. The final questionnaire contained a total of 89 items. Three key domains were identified to construct the questionnaire, which included image-based sexual abuse, nonimage-based TFSVA, and online-initiated physical sexual violence. The overall scale CVI values of relevance, appropriateness, and clarity for the scale were 0.90, 0.96, and 0.97, respectively, which indicated high content validity for all the instrument items. To ensure the measurement accurately reflects the experiences of diverse demographic groups, the content validity was further analyzed by gender and sexual orientation. This analysis revealed variations in item validity among participants from different genders and sexual orientations. For instance, heterosexual male respondents showed a particularly low CVI for relevance of 0.20 in the items related to nudity, including “male’s chest/nipples are visible” and “the person is sexually suggestive.” This underscored the importance of an inclusive approach when developing a measurement for TFSVA.

**Conclusions:**

This study greatly advances the assessment of TFSVA by examining the content validity of our newly developed measurement. The findings revealed that our measurement tool demonstrated adequate content validity, thereby providing a strong foundation for assessing TFSVA within the Chinese context. Implementing this tool is anticipated to enhance our understanding of TFSVA and aid in the development of effective interventions to combat this form of abuse.

## Introduction

### Background

The rapid advancement of technology has significantly transformed our modes of communication and interaction. While the digital era offers numerous benefits, it also presents heightened opportunities for online sexual violence, which has become increasingly prevalent worldwide [1-3]. Technology-facilitated sexual violence and abuse (TFSVA) refers to a spectrum of behaviors where digital technologies are used to facilitate both virtual and face-to-face sexual violence [4]. This insidious form of violence encompasses a wide range of harmful behaviors, such as online sexual harassment, cyberstalking, and image-based sexual abuse (IBSA) [4].

The use of technology, particularly the internet, can significantly increase the risk of sexual victimization [5]. Individuals who spend considerable time online may inadvertently expose themselves to potential abusers due to increased accessibility and visibility [6]. The association between technology and sexual abuse can be explained by 2 theories. First, according to O’Connell’s typology of online grooming, the ease of accessing potential victims online facilitates sexual abuse [7]. Historically, perpetrators commonly targeted individuals within their immediate social circles such as family, friends, or coworkers. However, the advent of the internet has significantly broadened the potential victim pool, making individuals more accessible to perpetrators [8]. Second, Suler’s theory of online disinhibition posits that the anonymity and perceived safety of online interactions encourage individuals to share personal information, trust strangers, and engage in risky behaviors more readily than they might in person [9]. People who are naturally kind, generous, and trusting are particularly vulnerable in these online environments [9]. These theories shed light on how the ease of accessing potential victims online and the anonymity of online interactions contribute to the vulnerability of individuals and facilitate sexual abuse in the digital age.

The prevalence of TFSVA is increasing as indicated by a meta-analysis and systematic review of 19 studies in 6 countries across the United States and Europe. The results of the pool prevalence showed that 8.8% of the general population had their images or video-based sexts distributed without permission, 7.2% had been threatened with sext data sharing, and 17.6% had their sexually explicit images taken without consent [10]. Additionally, the reported rates of engaging in TFSVA perpetration behaviors were estimated to be over 1 in 10 individuals [11].

Given the emergent status and escalating prevalence of TFSVA in today’s digital age, there is a critical need to explore the extent and nature of TFSVA across different populations [12,13]. The development of a standardized and comprehensive assessment instrument is of utmost importance to effectively measure this emerging and ever-changing phenomenon [14]. However, a significant hurdle is the lack of an agreed definition and clarity regarding TFSVA behaviors. A scoping review of 14 peer-reviewed studies across low- and middle-income countries revealed that different terms or concepts were used in the literature, for example, online sexual harassment, digital or online abuse, and technology-facilitated sexual violence [12]. Over half of the included studies did not state a clear definition of the terms being used [12].

Due to the lack of a precise definition of TFSVA, several limitations are identified among the existing measurements. In terms of comprehensiveness, many of the existing measurements have a narrow scope and only assess a single aspect of TFSVA, such as sexting, nonconsensual sharing of sexually explicit images, or online grooming [15-17]. TFSVA is a broad concept that should not be examined by a single behavior. In terms of uniformity, there is a large array of methods to examine a particular type of TFSVA. For instance, sexting behaviors have been widely studied, but the assessment methods and outcome measures have varied [15,18,19]. Inconsistent assessment methods can significantly affect the reliability and comparability of prevalence data across different studies [20]. In terms of cultural sensitivity, most studies have predominantly focused on Western populations [4]. This leaves a significant gap in the understanding of TFSVA in non-Western settings, particularly Asian settings, which represent a large portion of the global population [21]. Studying TFSVA in Asian contexts is crucial because the digital landscapes in many Asian countries are vast and distinct from those in the West, featuring unique social media platforms and digital communication tools that may influence the nature and prevalence of TFSVA differently [22]. Furthermore, cultural differences in attitudes toward privacy, gender roles, and sexual harassment may impact both the incidence of TFSVA and the willingness of survivors to report such incidents [23]. Additionally, the regulatory environments in various Asian countries, including internet censorship and social policies, could influence both the manifestation of TFSVA and the methodologies available for studying it [24]. Therefore, the development of a comprehensive, unified, and culturally sensitive measurement of TFSVA is needed.

### Aims and Objectives

Although the concepts of TFSVA are not well defined, TFSVA in this study refers to any form of sexual violence or harassment through the misuse of digital technologies [12]. The overall goal of this formative work is to develop a TFSVA measurement with sound content validity in the Chinese context. Since TFSVA is understudied in the Asian and Chinese contexts and there is no standardized or well-validated tool to assess TFSVA, the first objective of the study is to develop our own TFSVA instrument in traditional Chinese. The goal is to measure the intended concept and align it with the theoretical concept of TFSVA. The second objective of the study is to assess the content validity of the TFSVA items. Content validity evaluates the extent to which the measurement captures the specific content domain [25]. It determines whether the collection of sample items adequately defines the construct being measured [25]. Furthermore, it examines the relevance, representativeness, and comprehensiveness of the measurement instrument’s elements with regard to the intended assessment purpose [26].

## Methods

### Stage 1: Synthesis of Measurement Tools

The first step in measuring TFSVA victimization and perpetration was to conduct a thorough literature review of existing research on TFSVA and relevant measurement tools. A literature search was conducted by 2 independent researchers from database inception until June 2023 in English electronic databases, which included PubMed, Embase, and Scopus. The relevant question items were further reviewed by the principal investigator. The study included a selection of behaviors encompassed within the overarching definition of TFSVA, namely IBSA, nonimage-based technology-facilitated sexual violence and abuse (NIB-TFSVA), and online-initiated physical sexual violence (OIPSV). While the majority of existing empirical studies primarily focus on the first 2 forms, physical forms of sexual abuse facilitated by digital means, such as encounters arranged via dating apps that lead to forced sex, are often overlooked [14]. However, a qualitative study among gay dating app users in Hong Kong revealed that some individuals experience physical forms of sexual abuse with partners initially met through these apps [27]. This finding underscores the importance of including this domain in our questionnaire. Therefore, 3 key domains related to TFSVA were identified and served as the foundation for the synthesis of questionnaire items.

#### IBSA Category

IBSA is characterized by abuse involving the survivor’s visual representation without their consent. This form of abuse involves the unauthorized creation, distribution, or threat of sharing the intimate or sexual images of an individual [28]. Common examples are revenge porn and nonconsensual sharing of intimate photos. Fourteen items from a study conducted in Australia were used as key references for developing IBSA measurements [11]. Additionally, given the emergence of artificial intelligence (AI) technology, the phenomenon of “deepfake” has become more accessible and sophisticated in recent years [29]. Deepfake involves the use of AI to create realistic fake videos or images [29]. To address this emerging issue, 2 items about digitally altered images or videos and nonconsensual sexual deepfakes using AI were added. This domain emphasizes a type of abuse that is perpetrated directly through visual content, highlighting significant breaches of privacy and autonomy via the unauthorized use of the survivor’s image.

Eventually, 16 items related to IBSA victimization and 17 items related to IBSA perpetration, including scenarios, such as up-skirting, and situations where an individual is completely nude, were compiled for this study. To assess IBSA victimization, binary questions asked about whether any other individuals had taken, distributed, or threatened to distribute an image or a video without consent. If they reported experiencing the distribution of an image or a video without their consent, they were then asked to indicate the platforms on which it was shared, including social media, dating apps, mobile messaging platforms, and other online platforms. Regarding IBSA perpetration, questions asked about whether individuals had taken, distributed, forwarded, or threatened to distribute an image or a video of someone without their consent.

#### NIB-TFSVA Category

This broad category covers sexually abusive behaviors that are conducted through technological means and digital communication, rather than visual representation [12]. It includes but is not limited to actions like sending unsolicited sexually explicit messages (also known as “sexting”), cyberstalking, or coercing someone for sexual favors online [12]. The question items are derived from a study that focused on TFSVA among adults in Australia [30]. The TFSVA measurement used in the study covers several facets such as digital sexual harassment, sexual aggression and coercion, and gender or sexuality-based harassment. While this domain may involve scenarios with images, such as receiving or being pressured to send sexually explicit images, the primary form of abuse stems from coercive communication and manipulative interactions rather than the mere presence of images.

Finally, 19 items related to NIB-TFSVA were generated for this study. To understand the context in which these abusive acts occurred, binary questions asked whether individuals had experienced the mentioned incidents on social media, dating apps, mobile messaging platforms, and other online platforms. The same set of items was also adapted to assess the prevalence of TFSVA perpetration.

#### OIPSV Category

This category considered the physical form of sexual violence that stems from an initial online encounter, which could happen when perpetrators use technology to connect and further arrange meetings offline where an act of in-person sexual violence can occur [14]. Items from the sexual coercion subscale of the Revised Conflict Tactics Scale 2 (CTS-2) were used as the reference [31], with modifications to specifically ask about such experiences with people who were met online. Additionally, items, such as the nonconsensual removal of a condom, a practice known as “stealthing,” were included.

Eventually, 9 items were generated to measure this form of sexual violence. To assess victimization, questions asked if individuals had experienced the incidents described, such as being forced to have sex or condomless sex. Similarly, to measure perpetration, questions asked if individuals had done such acts to others. The frequency of occurrence of each act during the past year was measured on a 7-point Likert scale (0=never happened before; 1=once; 2=twice; 3=3 to 5 times; 4=6 to 10 times; 5=11 to 20 times; 6=more than 20 times). In addition to the 7-point Likert scale, there was a response option to indicate that the act did not occur in the past year but did happen before.

A total of 89 items were included in the questionnaire, with 33 items in the IBSA victimization and perpetration subscales, 38 items in the NIB-TFSVA victimization and perpetration subscales, and 18 items in the OIPSV victimization and perpetration subscales. As the question items were derived from English questionnaires, a translation process to traditional Chinese was performed and discrepancies were checked by 2 independent researchers. The principal investigator did a final cross-check of the translated versions.

### Stage 2: Judgement and Quantification of the Questionnaire

#### Expert Panel

After the initial generation of items, all the items were reviewed by an expert panel to assess the face validity. A group of experts from related fields were invited to review the questionnaire. They consisted of sexual health experts, nurses, and frontline workers experienced in sexual health education in the community.

The purpose of this expert review was to ensure the generated items accurately and sufficiently captured the intended constructs of TFSVA. The expert panel provided feedback on the wording, clarity, and appropriateness of the items. Based on their inputs, some items were refined or modified. This expert review process helped establish the face validity of the items, ensuring they were well suited for assessing the prevalence and characteristics of TFSVA experiences among the study participants.

#### Lay Panel

The next stage involved testing the questionnaire with potential research participants. The content validity of the instrument can be determined using the viewpoints of a panel of lay experts [32]. To assess the content validity of the items generated, the lay experts were asked to provide their judgments of the items [32]. Both qualitative and quantitative viewpoints on relevance, appropriateness, and clarity were collected to ensure the content validity of the items generated. The viewpoints of the potential study participants were quantified by computing the content validity index (CVI) [25].

#### Procedure for Stage 2

This stage involved a cross-sectional study that was conducted using an online questionnaire platform. Eligibility criteria required participants to be adults aged 18 or older who were proficient in Chinese. Participants were excluded if they refused to participate or were unable to provide consent.

The recruitment process began with convenience sampling through local nongovernmental organizations in Hong Kong that specialize in sexual health. Information about the study was distributed through online platforms, such as social media and email. Participants were recruited online. Subsequently, snowball sampling was employed, wherein enrolled participants were encouraged to invite friends who might be interested in joining the study.

### Measurement of Content Validity

Participants were asked to provide their viewpoints on the TFSVA questionnaire by rating each item in terms of its relevance, appropriateness, and clarity to the topic. The CVI was calculated for all individual items (I-CVI) and the overall scale (S-CVI). A 5-point Likert scale was used along the item rating continuum: 1=not relevant, 2=somewhat relevant, 3=neutral, 4=quite relevant, and 5=highly relevant. The same rating scale was used to assess the domains related to the appropriateness and clarity of the questionnaire. Additionally, participants could offer further comments or suggestions for improvement on any of the items.

### Data Analysis

The I-CVI was computed for each item as the number of participants providing a rating of 4 or 5, divided by the total number of participants. The acceptable value of I-CVI for more than 5 experts is 0.78 [25]. Therefore, a calculated I-CVI of 0.79 or more indicates the items are appropriate and retained, while 0.70 to 0.79 indicates the items need revisions. In contrast, a value less than 0.70 suggests that the items need to be eliminated.

The S-CVI was computed to ensure the content validity of the overall scale [33]. There are 2 types of S-CVI, including average S-CVI and overall S-CVI. The average S-CVI was calculated by adding all the I-CVI values and dividing by the number of items in the subscale [32]. On the other hand, the overall S-CVI was calculated by summing all average S-CVI values and dividing by the number of subscales [32]. It is recommended that the minimum S-CVI value should be 0.8 to reflect content validity [25,34]. Qualitative comments provided by the study participants were reviewed by the investigation team.

### Ethical Considerations

The study was approved by the Institutional Review Board of the University of Hong Kong/Hospital Authority Hong Kong West Cluster (HKU/HA HKW IRB; reference number: UW 23-397). Informed consent was obtained from each study participant. All study results presented in this paper were anonymous to protect the privacy and confidentiality of the participants.

## Results

### Overview

The study included responses from 24 lay experts, with a mean age of 27.96 (SD 4.27) years. In terms of gender, 15 (63%) were male and 9 (37%) were female. In terms of sexual orientation, 11 (46%) were heterosexual, 1 (4%) was bisexual, and 12 (50%) were homosexual. In terms of education level, 23 (96%) obtained a bachelor’s degree or above and 1 (4%) had a secondary school, diploma, or associate degree or below.

The overall S-CVI values of relevance, appropriateness, and clarity for the scale were 0.90, 0.96, and 0.97, respectively. The I-CVI values of the relevance, appropriateness, and clarity dimensions ranged from 0.71 to 1, 0.88 to 1, and 0.88 to 1, respectively. The results indicated high content validity of the instrument items. All items were included without further amendments in the final instrument (Multimedia Appendix 1).

### IBSA Category

The S-CVI values of relevance, appropriateness, and clarity for the IBSA victimization subscale were 0.92, 0.96, and 0.96 respectively. The S-CVI values of relevance, appropriateness, and clarity for the IBSA perpetration subscale were 0.89, 0.97, and 0.97, respectively. All items showed high I-CVI values in the dimensions of appropriateness and clarity, while some items showed low I-CVI values in the relevance dimension. There was 1 item with a relatively low I-CVI (relevance) of 0.79 in both the victimization and perpetration subscales, which was “you are sexually suggestive (eg, wearing provocative clothing/underwear and having body language/posture).” There was another item in the perpetration subscale with a low I-CVI (relevance) of 0.71, which was “male’s chest breast/nipple is visible.” A few participants queried whether the items related to up-skirting and visible bras were for females only. The results are shown in Tables 1 and 2.

**Table 1:** Content validity index of the items related to image-based sexual abuse victimization.

Item	Individual item content validity index^a^		
	Relevance	Appropriateness	Clarity
1. You are partially clothed or seminude (您穿著部分衣服或半裸著身體)	0.88	0.96	0.88
2. Your breasts/chests/cleavage/nipples are visible (可以看見您的乳房／胸部／乳溝，包括乳頭)	0.92	0.92	0.92
3. You are completely nude (您全裸著身體)	0.96	0.96	0.92
4. Your genitals are visible (可以看見您的性器官)	0.96	0.96	0.96
5. You engage in a sex act (您正在進行性行為)	0.92	0.96	1.00
6. You are showering, bathing, or toileting (您正在淋浴、浸浴或上廁所)	0.92	0.96	1.00
7. Presenting a sex act that you did not agree to (展示您不同意參與的性行為)	0.92	0.96	0.96
8. Images or videos are taken up your skirt (“up-skirting”) (是您裙底的位置（如：透過裙底向上拍/偷拍裙底）)	0.96	1.00	1.00
9. You are sexually suggestive (eg, wearing provocative clothing/underwear and having body language/posture) (你呈現出性暗示（例如：穿著挑逗性的服裝/內衣，以及身體語言/姿勢）)	0.79	0.96	0.96
10. Your underpants are visible (可以看見您的內褲)	0.88	0.92	0.96
11. The outline of your genital area (vagina/penis) is visible (可以看見您的生殖器/性器官 (如：陰部/陰莖) 的輪廓)	0.92	0.96	0.96
12. It makes you feel sexually offended or sexually violated (讓您感到性騷擾)	0.88	0.92	1.00
13. You are changing (您正在更衣)	0.96	0.96	0.96
14. Your bra is visible (可以看見您的胸圍)	0.88	0.88	0.92
15. Digitally altered images or videos that depict you in a sexual way (such as those created using Photoshop or other editing software) (經過數碼修改的照片或影片，呈現您帶有性意味的形象（例如使用 Photoshop 或其他編輯軟體所製作），包括移花接木等技術製作的虛假照片)	1.00	1.00	1.00
16. Nonconsensual sexual deepfakes (videos or images) created using deep learning artificial intelligence to replace, alter, or mimic your face or voice (使用深度學習人工智能技術（Deepfake）例如:替換、更改或模仿您的臉部或聲音，製作未經您同意的性深度偽造照片或影片)	1.00	1.00	1.00

^a^Average overall scale content validity index (S-CVI) values of relevance, appropriateness, and clarity were 0.92, 0.96, and 0.96, respectively.

**Table 2:** Content validity index of the items related to image-based sexual abuse perpetration.

Item	Individual item content validity index^a^		
	Relevance	Appropriateness	Clarity
1. The person is partially clothed or seminude (當事人穿著部分衣服或半裸著身體)	0.88	0.96	1.00
2a. Female’s breasts/nipples are visible (可以看見女性的胸部，包括乳頭)	0.92	1.00	1.00
2b. Male’s chests/nipples are visible (可以看見男性的胸部，包括乳頭)	0.71	0.92	0.96
3. The person is completely nude (當事人全裸著身體)	0.92	1.00	1.00
4. The person’s genitals are visible (可以看見當事人的性器官)	0.92	1.00	1.00
5. The person is engaged in a sex act (當事人正在進行性行為)	0.92	1.00	1.00
6. The person is showering, bathing, or toileting (當事人正在淋浴、浸浴或上廁所)	0.88	1.00	0.96
7. Presenting a sex act that the person did not agree to (展示當事人不同意參與的性行為)	0.92	0.96	0.96
8. Images or videos are taken up their skirt (“up-skirting”) (當事人裙底的位置（如：透過裙底向上拍/偷拍裙底）)	0.92	0.96	1.00
9. The person is sexually suggestive (eg, wearing provocative clothing/underwear and having body language/posture) (當事人呈現出性暗示（例如：穿著挑逗性的服裝/內衣，以及身體語言/姿勢）	0.79	1.00	1.00
10. The person’s underpants are visible (可以看見當事人的內褲)	0.88	0.96	0.96
11. The outline of the person’s genital area (vagina/penis) is visible (可以看見當事人的性器官 (如：陰部/ 陰莖)的輪廓)	0.92	0.96	0.96
12. The person might feel sexually offended or sexually violated (當事人可能會感到被性騷擾)	0.92	0.96	0.96
13. The person is changing (當事人正在更衣)	0.88	0.96	0.96
14. The person’s bra is visible (可以看見當事人的胸圍)	0.92	0.96	0.96
15. Digitally altered images or videos that depict another person in a sexual way (such as those created using Photoshop or other editing software) (經過數碼修改的照片或影片，呈現當事人帶有性意味的形象（例如使用 Photoshop 或其他編輯軟體所製作），包括移花接木等技術製作的虛假照片)	0.92	0.88	0.92
16. Nonconsensual sexual deepfakes (videos or images) created using deep learning artificial intelligence to replace, alter, or mimic another person’s face or voice (使用深度學習人工智能技術，例如替換、更改或模仿當時人的臉部或聲音，製作未經當事人同意的性深度偽造照片或影片)	0.92	0.96	0.96

^a^Average overall scale content validity index (S-CVI) values of relevance, appropriateness, and clarity were 0.89, 0.97, and 0.97, respectively.

### NIB-TFSVA Category

The S-CVI values of relevance, appropriateness, and clarity for the NIB-TFSVA victimization subscale were 0.91, 0.95, and 0.96, respectively. The S-CVI values of relevance, appropriateness, and clarity for the NIB-TFSVA perpetration subscales were 0.87, 0.97, and 0.98, respectively. All items in the victimization and perpetration subscales showed high I-CVI values, and participants did not have further comments related to the subscale items. The results are presented in Tables 3 and 4.

**Table 3:** Content validity index of the items related to nonimage-based technology-facilitated sexual violence and abuse victimization.

Item	Individual item content validity index^a^		
	Relevance	Appropriateness	Clarity
1. Receiving unwanted sexually explicit images or videos (收到不想要的色情或帶有性暗示的照片或影片)	0.96	0.96	1.00
2. Receiving unwanted sexually explicit comments or texts (收到不想要的色情或帶有性暗示的評論或短訊)	0.96	0.96	0.96
3. Receiving unwanted sexual requests (收到不想要的性請求)	0.96	1.00	0.92
4. Publicly posting online with offensive sexual comments about you (被人在網上公開發佈對您帶有性侮辱/性冒犯的評論)	0.92	1.00	0.96
5. Publicly posting online with personal details and/or pictures saying you are available to have sex (被人在網上公開發佈您的個人詳細資料和／或照片，聲稱您可以提供性服務／可以和其他人發生性關係)	0.92	1.00	1.00
6. Publicly posting online with personal details and/or pictures saying someone wants to have sex with you (被人在網上公開發佈您的個人詳細資料和／或照片，聲稱有人想和您發生性關係)	0.88	0.96	0.96
7. Having an unwanted sexual experience with someone met online (與網上認識的人有不想要／不願意的性經歷)	0.88	0.96	0.92
8. Receiving or being posted offensive and/or degrading messages, comments, or other content about your gender identity (收到或被張貼針對您性別認同的帶有冒犯性和／或貶低、侮辱意味的訊息、評論或其他內容)	0.88	0.92	0.92
9. Receiving or being posted offensive and/or degrading messages, comments, or other content about your sexual orientation (收到或被張貼針對您性傾向的帶有冒犯性和/或貶低、侮辱意味的訊息、評論或其他內容)	0.88	0.92	0.92
10. Receiving or being posted offensive and/or degrading messages, comments, or other content about your sex roles (收到或被張貼針對您性行為角色的帶有冒犯性和/或貶低、侮辱意味的訊息、評論或其他內容)	0.88	0.92	0.92
11. Receiving sexually violent threats, such as threats to rape you (收到性暴力的威脅，例如要強姦您)	0.88	0.96	0.92
12. Describing or visually representing an unwanted sexual act against you (被人以言語、圖像或其他視覺方式描述對您進行您不想要或不願意的性行為)	0.92	0.96	1.00
13. Being pressured to engage in phone sex (被逼進行電話性愛)	0.92	0.96	1.00
14. Being pressured to engage in sexual activity via chat room or video call (被逼通過聊天室或視像通話進行性行為)	0.92	0.92	0.96
15. Being pressured to engage in sexual acts on a digital device (eg, mobile phone, tablet, or computer) (被逼在電子設備（例如手機、平板或電腦）上進行性行為)	0.92	0.92	0.96
16. Being pressured to discuss sex-related topics on a digital device (eg, mobile phone, tablet, or computer) (被逼在電子設備（例如手機、平板或電腦）上討論性話題)	0.92	0.96	0.96
17. Being pressured to send nude images or videos on a digital device (eg, mobile phone, tablet, or computer) (被逼發送自己的裸體照片或影片)	0.88	0.88	1.00
18. Being pressured to send sexually explicit messages on a digital device (eg, mobile phone, tablet, or computer) (被逼在電子設備（例如手機、平板或電腦）上發送含有性暗示的訊息)	0.92	0.96	1.00
19. Your personal information and/or pictures are used without your consent to create a fake account for sexual purposes, such as arranging sexual hookups, sending sexual requests to others, and engaging in sexting (您的個人資料和/或照片被盜用於開設假帳戶去從事與性有關的活動，例如約別人進行性行為、向他人發送性請求和發送與性相關的短訊)	0.92	1.00	1.00

^a^Average overall scale content validity index (S-CVI) values of relevance, appropriateness, and clarity were 0.91, 0.95, and 0.96, respectively.

**Table 4:** Content validity index of the items related to nonimage-based technology-facilitated sexual violence and abuse perpetration.

Item	Individual item content validity index^a^		
	Relevance	Appropriateness	Clarity
1. Sending unsolicited sexually explicit images or videos (未經當事人同意，擅自發送色情或帶有性暗示的照片或影片)	0.88	0.92	0.96
2. Sending unsolicited sexually explicit comments or texts (未經當事人同意，擅自發送色情或帶有性暗示評論或短訊)	0.92	0.96	0.96
3. Sending unsolicited sexual requests (未經當事人同意發出性請求)	0.92	0.96	0.96
4. Publicly posting offensive sexual comments about others online (在網上公開發佈對別人帶有性侮辱／性冒犯的評論)	0.92	1.00	1.00
5. Publicly posting personal details and/or pictures of a person online, indicating that the person is offering sex service or is available for sex (在網上公開發佈別人的個人資料和／或照片，聲稱當事人可以提供性服務或可以與他人發生性關係)	0.92	1.00	0.96
6. Publicly posting personal details and/or pictures of a person online, indicating that you/someone wants to have sex with that person (在網上公開發佈別人的個人資料和／或照片，聲稱您或有人想和當事人發生性關係)	0.88	1.00	1.00
7. Forcing someone you met online to have sex with you (強逼您在網上認識的人與您發生性行為)	0.92	0.92	0.96
8. Sending or posting offensive and/or degrading messages, comments, or other content about others’ gender identity (發送或張貼針對當事人性別認同的帶有冒犯性和／或貶低、侮辱意味的訊息、評論或其他內容)	0.92	1.00	1.00
9. Sending or posting offensive and/or degrading messages, comments, or other content about others’ sexual orientation (發送或張貼針對當事人性傾向的帶有冒犯性和／或貶低、侮辱意味的訊息、評論或其他內容)	0.92	1.00	1.00
10. Sending or posting offensive and/or degrading messages, comments, or other content about others’ sex roles (發送或張貼針對當事人性行為角色的帶有冒犯性和／或貶低、侮辱意味的訊息、評論或其他內容)	0.92	1.00	1.00
11. Sending sexually violent threats, such as threats to rape others (發送性暴力威脅，例如威脅要強姦別人)	0.92	1.00	1.00
12. Describing or visually representing an unwanted sexual act against others (以言語、圖像或其他視覺方式描述或呈現對當事人進行其不想要的性行為)	0.02	1.00	1.00
13. Putting pressure on others to engage in phone sex (強逼當事人進行電話性愛)	0.92	0.92	0.96
14. Putting pressure on others to engage in sexual activity via chat room or video call (強逼當事人通過聊天室或視像通話進行性行為)	0.92	0.88	0.96
15. Putting pressure on others to engage in sexual acts on a digital device (eg, mobile phone, tablet, or computer) (逼當事人在電子設備（例如手機、平板或電腦）上進行性行為)	0.92	0.92	0.96
16. Putting pressure on others to discuss sex-related topics on a digital device (eg, mobile phone, tablet, or computer) (強逼當事人在電子設備（例如手機、平板或電腦）上討論性話題)	0.92	0.96	1.00
17. Putting pressure on others to send nude images or videos of himself or herself on a digital device (eg, mobile phone, tablet, or computer) (強逼當事人發送自己的裸體照片或影片)	0.92	0.96	1.00
18. Putting pressure on others to send sexually explicit messages on a digital device (eg, mobile phone, tablet, or computer) (強逼當事人在電子設備（例如手機、平板或電腦）上發送有性暗示的訊息)	0.92	0.96	1.00
19. Using others’ personal information and/or pictures without their consent to create a fake account for sexual purposes, such as arranging sexual hookups, sending sexual requests to others, and engaging in sexting (盜用別人的個人資料和／或照片，用於開設假帳戶去從事與性有關的活動，例如約別人進行性行為、向他人發送請求和發送與性相關的短訊)	0.92	1.00	1.00

^a^Average overall scale content validity index (S-CVI) values of relevance, appropriateness, and clarity were 0.87, 0.97, and 0.98.

### OIPSV Category

The S-CVI values of relevance, appropriateness, and clarity for the OIPSV victimization subscale were 0.95, 0.98, and 0.98, respectively. The S-CVI values of relevance, appropriateness, and clarity for the OIPSV perpetration subscale were 0.85, 0.93, and 0.93, respectively. All items had high I-CVI values, and participants did not have additional comments on the subscale items. The results are presented in Tables 5 and 6.

**Table 5:** Content validity index of the items related to online-initiated physical sexual violence victimization.

Item	Individual item content validity index^a^		
	Relevance	Appropriateness	Clarity
1. Insisting on having sex with you (but did not use physical force) (在沒有使用武力的情況下，對方堅持與您發生性行為)	0.92	1.00	1.00
2. Using threats to force you to have sex (but did not use physical force) (在沒有使用武力的情況下，對方以威嚇來強逼您與對方本人發生性行為)	0.96	1.00	0.96
3. Using physical force (such as hitting, holding down, or using a weapon) to force you to have sex (以武力（例如打您、按住您、或使用武器）來強逼您與對方本人發生性行為)	0.96	0.92	0.96
4. Insisting on having condomless sex with you (but did not use physical force) (在沒有使用武力的情況下，對方堅持要與您進行無套的性行為)	0.96	1.00	1.00
5. Using threats to force you to have condomless sex (but did not use physical force) (在沒有使用武力的情況下，對方以威嚇來強逼您與對方本人發生無套的性行為)	0.92	0.96	0.96
6. Using physical force (such as hitting, holding down, or using a weapon) to force you to have condomless sex (以武力（例如打您、按住您、或使用武器）來逼您進行無套的性行為)	0.96	1.00	1.00
7. Nonconsensual condom removal during sexual activity (also known as “stealthing”) (性行為期間，在您不知情的情況下把安全套脫掉)	0.92	1.00	1.00
8. Ejaculating in/on your body without your consent (沒有您的同意下，在您的身體内或表面射精)	0.96	0.96	0.96
9. Intentionally transmitting HIV or other STIs to you (故意將愛滋病／其他性病傳染給您)	0.96	1.00	0.96

^a^Average overall scale content validity index (S-CVI) values of relevance, appropriateness, and clarity were 0.95, 0.98, and 0.98.

**Table 6:** Content validity index of the items related to online-initiated physical sexual violence perpetration.

Item	Individual item content validity index^a^		
	Relevance	Appropriateness	Clarity
1. When meeting people online, have you ever insisted on having sex with others (but did not use physical force) (與在網上認識的人見面時，**您**有沒有曾經**在沒有使用武力的情況下，堅持與對方發生性行為)**	0.88	0.96	0.96
2. When meeting people online, have you ever used threats to force others to have sex (but did not use physical force) (與在網上認識的人見面時，**您**有沒有曾經在沒有使用武力的情況下，以威嚇來強逼對方與您發生性行為)	0.83	0.88	0.88
3. When meeting people online, have you ever used physical force (such as hitting, holding down, or using a weapon) to force others to have sex (與在網上認識的人見面時，**您**有沒有曾經以武力（例如打對方、按住對方、或使用武器）來強逼對方與您發生性行為)	0.82	0.92	0.92
4. When meeting people online, have you ever insisted on having condomless sex with others (but did not use physical force) (與在網上認識的人見面時，**您**有沒有曾經**在**沒有使用武力的情況下，堅持要與對方進行無套的性行為)	0.82	0.92	0.92
5. When meeting people online, have you ever used threats to force others to have condomless sex (but did not use physical force) 與在網上認識的人見面時，**您**有沒有曾經在沒有使用武力的情況下，以威嚇來強逼對方與您發生無套的性行為)	0.82	0.92	0.92
6. When meeting people online, have you ever used physical force (such as hitting, holding down, or using a weapon) to force others to have condomless sex (與在網上認識的人見面時，**您**有沒有曾經以武力（例如打**對方**、按住**對方**、或使用武器）來逼**對方**進行無套的性行為)	0.88	0.96	0.96
7. When meeting people online, have you ever removed the condom during sexual activity without their consent (also known as “stealthing”) (與在網上認識的人見面時，**您**有沒有曾經**在**性行為期間，在**對方**不知情的情況下把安全套脫掉)	0.88	0.96	0.96
8. When meeting people online, have you ever ejaculated in/on others’ bodies without their consent (與在網上認識的人見面時，**您**有沒有曾經**在**沒有**對方**的同意下，在**對方**的身體内或表面射精)	0.88	0.96	0.96
9. When meeting people online, have you ever intentionally transmitted HIV or other STIs to others (與在網上認識的人見面時，**您**有沒有曾經**故意將愛滋病病毒／其他性病傳染給對方)**	0.82	0.92	0.92

^a^Average overall scale content validity index (S-CVI) values of relevance, appropriateness, and clarity were 0.85, 0.93, and 0.93, respectively.

### Content Validity Assessed by Gender and Sexual Orientation

The content validity of the measurement was further assessed by gender and sexual orientation, categorizing participants into 4 groups: heterosexual males (5/24, 21%), bisexual or gay males (10/24, 42%), heterosexual females (6/24, 25%), and bisexual or lesbian females (3/24, 12%). The results are shown in Multimedia Appendix 2.

In the subscale related to IBSA victimization (Table S1 in Multimedia Appendix 2), heterosexual male respondents showed a low average S-CVI (relevance) of 0.73. The items “you are partially clothed or semi-nude,” “you are engaged in a sex act,” “you are sexually suggestive,” “your underpants/bras are visible,” and “it makes you feel sexually offended or sexually violated” showed I-CVI (relevance) values lower than 0.8. Additionally, heterosexual female respondents had a low I-CVI (relevance) of 0.67 for the item “you are sexually suggestive.” They also showed a low I-CVI (appropriateness and clarity) of 0.67 for the item “your bra is visible.” Other groups of respondents showed acceptable S-CVI and I-CVI values for the dimensions related to relevance, appropriateness, and clarity in the IBSA victimization subscale.

In the subscale related to IBSA perpetration (Table S2 in Multimedia Appendix 2), heterosexual male respondents showed a low average S-CVI (relevance) of 0.69. Two items (“male’s chest/nipples are visible” and “the person is sexually suggestive”) showed a particularly low I-CVI (relevance) of 0.20. Heterosexual female respondents showed a low I-CVI (appropriateness and clarity) of 0.67 for the item “digitally altered images or videos that depict another person in a sexual way.” The remaining groups of respondents showed acceptable S-CVI and I-CVI values for the dimensions related to relevance, appropriateness, and clarity in the subscale.

In the subscales related to NIB-TFSVA victimization (Table S3 in Multimedia Appendix 2), heterosexual female respondents showed a low average S-CVI (relevance) of 0.78. Items with low I-CVI values in all dimensions included “receiving or being posted offensive and/or degrading messages, comments, or other content about your gender identity, sexual orientation, and sex roles.” The remaining groups of respondents showed acceptable S-CVI and I-CVI values in all the dimensions.

In the subscales related to NIB-TFSVA perpetration (Table S4 in Multimedia Appendix 2), all respondents showed high average S-CVI values in all dimensions. Heterosexual female respondents showed low I-CVI (relevance) values for the items “sent unsolicited sexually explicit images or videos” and “publicly posted personal details and/or pictures of a person online, indicating that someone wants to have sex with that person.” Heterosexual male respondents showed low I-CVI (appropriateness) values for the items “sent unsolicited sexually explicit comments or texts” and “forced someone you met online to have sex with you.”

In the subscales related to OIPSV victimization (Table S5 in Multimedia Appendix 2), heterosexual male respondents showed a low average S-CVI (relevance) of 0.78. The I-CVI (relevance) of the item “nonconsensual condom removal during sexual activity” was 0.60. Heterosexual female respondents showed a low I-CVI (appropriateness) of 0.67 for the item “using physical force to force you to have sex.”

In the subscales related to OIPSV perpetration (Table S6 in Multimedia Appendix 2), both heterosexual male and female respondents showed low average S-CVI (relevance) values of 0.60 and 0.74, respectively. Heterosexual female respondents also appeared to have a low average S-CVI of 0.72 in the dimensions of appropriateness and clarity. Items with low I-CVI values for all dimensions among heterosexual male and female respondents included “used threats to force others to have sex but did not use physical force,” “used physical force to force others to have sex,” “insisted on having condomless sex with others but did not use physical force,” “used threats to force others to have condomless sex but did not use physical force,” and “intentionally transmitted HIV or other STIs to others.”

## Discussion

### Principal Findings

This study aimed to develop a TFSVA measurement tool with robust content validity in the Chinese context. Three key domains were identified to construct the questionnaire, which included IBSA, NIB-TFSVA, and OIPSV. The final questionnaire contained a total of 89 items, and all the instrument items showed a high content validity. The content validity was further analyzed by gender and sexual orientation to ensure the measurement accurately reflected the experiences of diverse demographic groups. This analysis revealed variations in item validity among participants from different genders and sexual orientations, underscoring the importance of an inclusive approach for scale development.

TFSVA is a relatively new and complex concept, and it encompasses a diverse range of abusive acts [4]. As technology continues to advance, new forms of abuse that were previously unimaginable are beginning to surface [4]. For instance, the rapid development of AI has given rise to concerns such as deepfakes and digitally altered sexually explicit imagery [35]. Given the dynamic and ever-evolving nature of TFSVA, establishing a unified and inclusive definition poses significant challenges [20]. This lack of a standardized definition further complicates the creation of comprehensive and standardized instruments to measure this multifaceted phenomenon [20]. The constant evolution of technology means that TFSVA can manifest in forms today that were not considered previously, making it exceedingly difficult to capture the full spectrum of abusive behaviors in a single and cohesive framework [4]. This situation underscores the necessity for ongoing research and the adaptation of measurement tools to keep pace with technological advancements and emerging trends in sexual abuse.

We acknowledge that, unlike other well-defined constructs, such as depression, anxiety, and health-related quality of life, no universally standardized instrument comprehensively covers all aspects of TFSVA. Existing instruments of TFSVA vary widely in terms of the items and concepts they measure [4,10,36]. In light of this, our focus was on crafting an instrument that, while not exhaustive, is sufficiently comprehensive to address the key aspects of TFSVA relevant to the Chinese context. This approach ensures that the questionnaire is both practical for our study and sensitive to the specific nuances of the population we are examining.

Furthermore, results from the content validity assessment illuminate how participants’ interpretations of TFSVA may differ significantly based on their gender and sexual orientation. For instance, heterosexual male participants found items, like exposing the male chest and underpants and showing someone bathing or toileting, irrelevant to TFSVA, while gay or bisexual male participants and all female participants found these items relevant to the topic. This shows that the attitudes and perceptions toward sexual abuse and nudity can vary greatly among individuals with different genders and sexual orientations. These differences in perceptions could be shaped by cultural, social, and individual factors.

In terms of social and cultural factors, society often sexualizes the female body to a greater extent than the male body, which can lead to the perception that female nudity or exposure is more sexually offensive compared to male nudity [37]. In addition, women’s bodies have been subjected to more scrutiny, which can influence perceptions of exposure [38]. These kinds of sexual objectifications and gendered norms can contribute to the differing reactions between men and women in viewing nudity and physical exposure.

In terms of individual factors, sexual orientation may influence how sexual abuse behaviors and physical exposure are perceived. For instance, heterosexual men may view female nudity as aligning with their sexual attractions and desires, potentially leading to a different response compared to gay men and women [39]. The alignment of personal attraction with the displayed gender can affect the perceived sexual nature of the situation [39]. In addition, individuals’ perspectives and experiences are shaped by their unique backgrounds, identities, and social contexts [40]. Therefore, people can have different viewpoints on TFSVA behaviors based on their personal experiences and beliefs.

### Strengths and Limitations

Our study is pioneering in that it is the first to explore and provide information about the content validity of a TFSVA questionnaire. Content validity is crucial as it ensures that the questionnaire items are relevant, clear, and appropriately tailored to the target audience [41]. This aspect of measurement development is particularly critical in surveys addressing sensitive subjects like sexual abuse, where participants often self-administer the questionnaire. Inaccuracies or ambiguities in the wording of questions could lead to misinterpretations, thereby affecting the reliability and validity of the data collected [42]. Moreover, due to the sensitive nature of the topic, ensuring that the questions are formulated with sensitivity and respect is essential to encourage honest and thoughtful responses from participants [43]. The careful design of these items also helps to minimize any potential distress caused by recalling or discussing personal experiences of abuse [43].

Another notable contribution of our study is the development of a questionnaire that assesses both perpetration and victimization in cases of TFSVA. This dual focus sets our work apart from many previous studies, which typically concentrated on either perpetration or victimization but rarely both [30,44]. Understanding the dynamics of perpetration is essential for generating valuable data, which, although scarce, is crucial for informing preventative methods. While it is essential to prevent individuals from becoming victims or experiencing further victimization, it is equally important to deter potential perpetrators from engaging in abusive behaviors [45]. Capturing empirical data on the preparatory behaviors of perpetrators is imperative before effective advocacy and intervention strategies can be developed [45]. Our study, therefore, fills a critical gap by offering a comprehensive view that includes these preparatory actions, which are often overlooked in research focused solely on victimization. This comprehensive approach allows for more effective development of prevention strategies that address all facets of TFSVA.

Another strength of our study lies in the design of our questionnaire, which was thoughtfully crafted to address TFSVA across a spectrum of gender and sexual orientations. Importantly, we ensured that the content validity of the questionnaire was rigorously evaluated for both gender and sexual orientation sensitivity. A mixed-methods study that examined the pathways of suicidal affect, cognition, and behavior within the context of TFSVA victimization supported the gender similarities hypothesis that TFSVA is not exclusively a gender-based harm [46]. Study results showed that TFSVA experiences and negative impacts are similar for both women and men [46]. This highlighted the need for greater awareness and increased support for all survivors. A wider range of items should be included to cater to the needs of respondents with different gender and sexual orientations.

Some limitations of the study should be noted. First, participant recruitment online may cause biases related to response or nonresponse, as those who choose to participate may systematically differ from those who choose not to [47]. This may lead to a higher risk of sampling bias, causing findings not to be generalizable to the general population [47]. Additionally, the data of bisexual and homosexual individuals were combined when assessing content validity by gender and sexual orientation, due to the small sample size (1 bisexual participant only). It was acknowledged that populations of different sexual orientations are not directly comparable. For instance, bisexual individuals experience a higher rate of sexual abuse compared with other groups [48]. Moreover, the content validity of the measurement in other Chinese contexts is uncertain as this study only recruited participants in Hong Kong. Considering cultural diversity, the current scale may not unambiguously represent all Chinese populations. Lastly, some scales, such as CTS-2, used in this paper were not developed for sexual minorities. However, this paper represents the first step in developing a comprehensive questionnaire, and the main purpose is to check the content validity of the questionnaire. To understand whether people with a different gender and sexual orientation would have different views on the question items, content validity was further assessed by gender and sexual orientation. The results showed that sexual minorities have different concerns when compared with heterosexual populations, which would be helpful for scale development in the future.

### Conclusion

This study contributes to the assessment of TFSVA by providing a thorough examination of the content validity of the questionnaire. The inclusion of CVI results and respondents’ comments helped establish the questionnaire’s content validity. Despite certain limitations, the findings support the questionnaire’s adequacy and relevance in measuring TFSVA. Future studies should further explore the psychometric properties and applicability of the questionnaire in different populations and cultural contexts to enhance its validity and utility. Lastly, it is necessary to periodically revisit and revise the instrument to ensure its relevance and accuracy as technology continues to evolve, potentially affecting the typology of TFSVA.

## Appendix

Multimedia Appendix 1 Questionnaire items.

Multimedia Appendix 2 Results of content validity assessed by gender and sexual orientation.

## Abbreviations

AI: artificial intelligence
CTS-2: Revised Conflict Tactics Scale 2
CVI: content validity index
IBSA: image-based sexual abuse
I-CVI: individual item content validity index
NIB-TFSVA: nonimage-based technology-facilitated sexual violence and abuse
OIPSV: online-initiated physical sexual violence
S-CVI: overall scale content validity index
TFSVA: technology-facilitated sexual violence and abuse

## Data Availability

The datasets generated during or analyzed during this study are available from the corresponding author upon reasonable request.
