# Supplementary material for: Measuring Technology-Facilitated Sexual Violence and Abuse in the Chinese Context: Development Study and Content Validity Analysis
Source: JMIR Form Res. 2024 Nov 19;8:e65199. doi: 10.2196/65199 (PMC11615559; doi:10.2196/65199)
Supplement: Multimedia Appendix 1 [file formative_v8i1e65199_app1.docx]

**Multimedia Appendix 1.** Questionnaire items.

Part A: Prevalence of Image-Based Sexual Violence (Victimization)

**Have you ever had a nude or sexual image/video being taken, distributed and/or threatened to be distributed without your consent?**

1. You are partially clothed or semi-nude.
2. Your breasts/chests, including your nipples, are visible
3. You are completely nude
4. Your genitals are visible
5. You are engaged in a sex act
6. You are showering, bathing or toileting
7. Presents a sex act that you did not agree to
8. It was taken up your skirt (‘up-skirting’)
9. You are sexually suggestive (e.g., wearing provocative clothing/ underwear and having body language/ posture)
10. Your underpants are visible
11. The outline of your genital area (vagina/ penis) is visible
12. It makes you feel sexually offended or sexually violated
13. You are changing
14. Your bra is visible
15. Digitally altered images or videos that depict you in a sexual way (such as those created using Photoshop or other editing software)
16. Non-consensual sexual deepfakes (videos or images) created using deep learning artificial intelligence to replace, alter, or mimic your face or voice

Part B: Prevalence of Non-Image-Based Technology-Facilitated Sexual Violence and Abuse (Victimization)

**Did the following incidents ever happen to you?**

1. Received unwanted sexually explicit images or videos
2. Received unwanted sexually explicit comments or texts
3. Received unwanted sexual requests
4. Being publicly posted online with offensive sexual comments about you
5. Being publicly posted online with personal details and/or pictures saying you are available to have sex
6. Being publicly posted online with personal details and/or pictures saying someone wants to have sex with you
7. Had an unwanted sexual experience with someone met online
8. Received or being posted offensive and/or degrading messages, comments, or other content about your gender identity.
9. Received or being posted offensive and/or degrading messages, comments, or other content about your sexual orientation
10. Received or being posted offensive and/or degrading messages, comments, or other content about your sex roles
11. Received sexually violent threats, such as threats to rape you
12. Described or visually represented unwanted sexual act against you
13. Being pressured to engage in phone sex
14. Being pressured to engage in sexual activity via chat room or video call
15. Being pressured to engage in sexual acts on a digital device (e.g., mobile phone, tablet or computer)
16. Being pressured to discuss sex-related topics on a digital device (e.g., mobile phone, tablet or computer)
17. Being pressured to send nude image(s) or video of myself
18. Being pressured to send sexually explicit messages on a digital device (e.g., mobile phone, tablet or computer)
19. Your personal information and/or pictures were used without your consent to create a fake account for sexual purposes, such as arranging sexual hookups, sending sexual requests to others, and engaging in sexting.

Part C: Prevalence of Image-Based Sexual Violence (Perpetration)

**Have you ever taken, distributed, forwarded and/or threatened to distribute a nude or sexual image/ video of another person (regardless of gender) without consent?**

1. The person was partially clothed or semi-nude
2. Female’s breasts, including the nipples, were visible
3. Male’s chests, including the nipples, were visible
4. The person was completely nude
5. The person’s genitals were visible
6. The person was engaged in a sex act
7. The person was showing bathing or toileting
8. Presents a sex act that the person did not agree to
9. Images or videos taken up their skirt (‘up-skirting’)
10. The person was sexually suggestive (e.g., wearing provocative clothing/ underwear and having body language/ posture)
11. The person’s underpants are visible
12. The outline of a person's genital area (vagina/penis) is visible
13. The person might feel sexually offended or sexually violated
14. The person was changing
15. The person’s bra is visible
16. Digitally altered images or videos that depict another person in a sexual way (such as those created using Photoshop or other editing software)
17. Non-consensual sexual deepfakes (videos or images) created using deep learning artificial intelligence to replace, alter, or mimic another person’s face or voice

Part D: Prevalence of Non-Image-Based Technology-Facilitated Sexual Violence and Abuse (Perpetration)

**Did you ever do any of the following things?**

1. Sent unsolicited sexually explicit images or videos
2. Sent unsolicited sexually explicit comments or texts
3. Sent unsolicited sexual requests
4. Publicly posted offensive sexual comments about others online
5. Publicly posted personal details and/or pictures of a person online, indicating that the person is offering sex service or is available for sex
6. Publicly posted personal details and/or pictures of a person online, indicating that you/ someone wants to have sex with that person
7. Forced someone you met online to have sex with you
8. Sent or posted offensive and/or degrading messages, comments, or other content about others’ gender identity
9. Sent or posted offensive and/or degrading messages, comments, or other content about others’ sexual orientation
10. Sent or posted offensive and/or degrading messages, comments, or other content about other’s sex roles
11. Sent sexually violent threats, such as threats to rape others
12. Described or visually represented an unwanted sexual act against others
13. Pressured others to engage in phone sex
14. Pressured others to engage in sexual activity via char room or video call
15. Pressured others to engage in sexual acts on a digital device (e.g., mobile phone, tablet or computer)
16. Pressured others to discuss sex- related topics on a digital device (e.g., mobile phone, tablet or computer)
17. Pressured others to send nude image(s) or video of himself or herself
18. Pressured others to send sexually explicit messages on a digital device (e.g., mobile phone, tablet or computer)
19. Used others’ personal information and/or pictures without their consent to create a fake account for sexual purposes, such as arranging sexual hookups, sending sexual requests to others, and engaging in sexting

Part E: Online-Initiated Physical Sexual Violence

**Have you experienced any of the following incidents when meeting people online, such as through dating apps, social media (e.g., Twitter and Instagram) or other online platforms?**

1. Insisting on having sex with you (but did not use physical force)

1a. You did this to others you met online

1. Using threats to force you to have sex (but did not use physical force)

2a. You did this to others you met online

1. Using physical force (such as hitting, holding down or using a weapon) to force you to have sex

3a. You did this to others you met online

1. Insisting on having condomless sex with you (but did not use physical force)

4a. You did this to others you met online

1. Using threats to force you to have condomless sex (but did not use physical force)

5a. You did this to others you met online

1. Using physical force (such as hitting, holding down or using a weapon) to force you to have condomless sex

6a. You did this to others you met online

1. Non-consensual condom removal during sexual activity, also known as 'stealthing'

7a. You did this to others you met online

1. Ejaculation in/on your body without your consent

8a. You did this to others you met online

1. Intentionally transmitting HIV/ or other STI to you?

9a. You did this to others you met online
