# Supplementary material for: Measuring Technology-Facilitated Sexual Violence and Abuse in the Chinese Context: Development Study and Content Validity Analysis
Source: JMIR Form Res. 2024 Nov 19;8:e65199. doi: 10.2196/65199 (PMC11615559; doi:10.2196/65199)
Supplement: Multimedia Appendix 2 [file formative_v8i1e65199_app2.docx]

**Multimedia Appendix 2.** Results of content validity assessed by gender and sexual orientation.

[Table S1. CVI of the items related to IBSA victimization 2](#_Toc177741148)

[Table S2. CVI of the items related to IBSA perpetration 4](#_Toc177741149)

[Table S3. CVI of the items related to NIB-TFSA victimization 7](#_Toc177741150)

[Table S4. CVI of the items related to NIB-TFSA perpetration 11](#_Toc177741151)

[Table S5. CVI of the items related to OIPSV victimization 15](#_Toc177741152)

[Table S6. CVI of the items related to OIPSV perpetration 17](#_Toc177741153)

**Note:**

HM: heterosexual male (n=5)

BM: biseuxal/gay male (n=10)

HF: heterosexual female (n=6)

BF: bisexual/lesbian female (n=3)

# Table S1. CVI of the items related to IBSA victimization.

| **Item** | **I-CVI** | | | | | | | | | | | |
| --- | --- | --- | --- | --- | --- | --- | --- | --- | --- | --- | --- | --- |
|  | **Relevance** | | | | **Appropriateness** | | | | **Clarity** | | | |
|  | **HM** | **BM** | **HF** | **BF** | **HM** | **BM** | **HF** | **BF** | **HM** | **BM** | **HF** | **BF** |
| 1. You are partially clothed or semi-nude (您穿著部分衣服或半裸著身體) | 0.40 | 1 | 1 | 1 | 0.80 | 1 | 1 | 1 | 0.80 | 0.90 | 0.83 | 1 |
| 2. Your breasts/chests/ cleavage/nipples, are visible (可以看見您的乳房／胸部／乳溝，包括乳頭) | 0.80 | 0.90 | 1 | 1 | 1 | 0.90 | 0.83 | 1 | 0.80 | 1 | 0.83 | 1 |
| 3. You are completely nude (您全裸著身體) | 0.80 | 1 | 1 | 1 | 1 | 1 | 0.83 | 1 | 0.80 | 1 | 0.83 | 1 |
| 4. Your genitals are visible (可以看見您的性器官) | 0.80 | 1 | 1 | 1 | 1 | 1 | 0.83 | 1 | 1 | 1 | 0.83 | 1 |
| 5. You are engaged in a sex act (您正在進行性行為) | 0.60 | 1 | 1 | 1 | 1 | 1 | 1 | 1 | 1 | 1 | 1 | 1 |
| 6. You are showering, bathing or toileting (您正在淋浴、浸浴或上廁所) | 0.80 | 1 | 0.83 | 1 | 0.80 | 1 | 1 | 1 | 1 | 1 | 1 | 1 |
| 7. Presents a sex act that you did not agree to (展示您不同意參與的性行為) | 0.80 | 1 | 0.83 | 1 | 0.80 | 0.90 | 1 | 1 | 0.80 | 0.90 | 1 | 1 |
| 8. It was taken up your skirt (‘up-skirting’) (是您裙底的位置（如：透過裙底向上拍/偷拍裙底）) | 0.80 | 1 | 1 | 1 | 1 | 1 | 1 | 1 | 1 | 1 | 1 | 1 |
| 9. You are sexually suggestive (e.g., wearing provocative clothing/ underwear and having body language/ posture) (你呈現出性暗示（例如：穿著挑逗性的服裝/內衣， 以及身體語言/姿勢）) | 0.40 | 1 | 0.67 | 1 | 0.80 | 1 | 1 | 1 | 0.80 | 1 | 1 | 1 |
| 10. Your underpants are visible (可以看見您的內褲) | 0.60 | 1 | 0.83 | 1 | 0.80 | 0.90 | 1 | 1 | 0.80 | 1 | 1 | 1 |
| 11. The outline of your genital area (vagina/ penis) is visible (可以看見您的生殖器/性器官 (如：陰部/陰莖) 的輪廓) | 0.80 | 1 | 0.83 | 1 | 1 | 1 | 0.83 | 1 | 1 | 1 | 0.83 | 1 |
| 12. It makes you feel sexually offended or sexually violated (讓您感到性騷擾) | 0.60 | 1 | 0.83 | 1 | 0.80 | 1 | 0.83 | 1 | 0.80 | 1 | 1 | 1 |
| 13. You are changing (您正在更衣) | 0.80 | 1 | 0.83 | 1 | 1 | 1 | 0.83 | 1 | 1 | 1 | 0.83 | 1 |
| 14. Your bra is visible (可以看見您的胸圍) | 0.60 | 0.90 | 0.83 | 1 | 1 | 0.90 | 0.67 | 1 | 1 | 1 | 0.67 | 1 |
| 15. Digitally altered images or videos that depict you in a sexual way (such as those created using Photoshop or other editing software)  (經過數碼修改的照片或影片，呈現您帶有性意味的形象 （例如使用 Photoshop 或其他編輯軟體所製作）， 包括移花接木等技術製作的虛假照片) | 1 | 1 | 1 | 1 | 1 | 1 | 1 | 1 | 1 | 1 | 1 | 1 |
| 16. Non-consensual sexual deepfakes (videos or images) created using deep learning artificial intelligence to replace, alter, or mimic your face or voice  (使用深度學習人工智能技術（Deepfake）例如:替換、  更改或模仿您的臉部或聲音，  製作未經您同意的性深度偽造照片或影片) | 1 | 1 | 1 | 1 | 1 | 1 | 1 | 1 | 1 | 1 | 1 | 1 |
| **Average S-CVI** | **0.73** | **0.99** | **0.91** | **1** | **0.93** | **0.98** | **0.92** | **1** | **0.91** | **0.99** | **0.92** | **1** |

# Table S2. CVI of the items related to IBSA perpetration.

| **Item** | **I-CVI** | | | | | | | | | | | |
| --- | --- | --- | --- | --- | --- | --- | --- | --- | --- | --- | --- | --- |
|  | **Relevance** | | | | **Appropriateness** | | | | **Clarity** | | | |
|  | **HM** | **BM** | **HF** | **BF** | **HM** | **BM** | **HF** | **BF** | **HM** | **BM** | **HF** | **BF** |
| 1. The person was partially clothed or semi-nude (當事人穿著部分衣服或半裸著身體) | 0.60 | 1 | 0.83 | 1 | 0.80 | 1 | 1 | 1 | 1 | 1 | 1 | 1 |
| 2a. Female’s breasts/ nipples were visible (可以看見女性的胸部，包括乳頭) | 0.80 | 1 | 0.83 | 1 | 1 | 1 | 1 | 1 | 1 | 1 | 1 | 1 |
| 2b. Male’s chests breasts/ nipples were visible (可以看見男性的胸部，包括乳頭) | 0.20 | 0.80 | 0.83 | 1 | 0.80 | 0.90 | 1 | 1 | 0.80 | 1 | 1 | 1 |
| 3. The person was completely nude (當事人全裸著身體) | 0.80 | 1 | 0.83 | 1 | 1 | 1 | 1 | 1 | 1 | 1 | 1 | 1 |
| 4. The person’s genitals were visible (可以看見當事人的性器官) | 0.80 | 1 | 0.83 | 1 | 1 | 1 | 1 | 1 | 1 | 1 | 1 | 1 |
| 5. The person was engaged in a sex act (當事人正在進行性行為) | 0.80 | 1 | 0.83 | 1 | 1 | 1 | 1 | 1 | 1 | 1 | 1 | 1 |
| 6. The person was showing bathing or toileting (當事人正在淋浴、浸浴或上廁所) | 0.60 | 1 | 0.83 | 1 | 1 | 1 | 1 | 1 | 0.80 | 1 | 1 | 1 |
| 7. Presents a sex act that the person did not agree to (展示當事人不同意參與的性行為) | 0.80 | 1 | 0.83 | 1 | 0.80 | 0.90 | 1 | 1 | 0.80 | 0.90 | 1 | 1 |
| 8. Images or videos taken up their skirt (‘up-skirting’) (當事人裙底的位置（如：透過裙底向上拍/偷拍裙底）) | 0.80 | 1 | 0.83 | 1 | 1 | 1 | 0.83 | 1 | 1 | 1 | 0.83 | 1 |
| 9. The person was sexually suggestive (e.g., wearing provocative clothing/ underwear and having body language/ posture) (當事人呈現出性暗示（例如：  穿著挑逗性的服裝/內衣，以及身體語言/姿勢） | 0.20 | 1 | 0.83 | 1 | 1 | 1 | 1 | 1 | 1 | 1 | 1 | 1 |
| 10. The person’s underpants are visible (可以看見當事人的內褲) | 0.60 | 1 | 0.83 | 1 | 0.80 | 1 | 1 | 1 | 0.80 | 1 | 1 | 1 |
| 11. The outline of a person's genital area (vagina/penis) is visible (可以看見當事人的性器官 (如：陰部/ 陰莖)的輪廓) | 0.80 | 1 | 0.83 | 1 | 1 | 1 | 0.83 | 1 | 1 | 1 | 0.83 | 1 |
| 12. The person might feel sexually offended or sexually violated (當事人可能會感到被性騷擾) | 0.80 | 1 | 0.83 | 1 | 0.80 | 1 | 1 | 1 | 0.80 | 0.90 | 1 | 1 |
| 13. The person was changing (當事人正在更衣) | 0.80 | 0.90 | 0.83 | 1 | 0.80 | 1 | 1 | 1 | 0.80 | 1 | 1 | 1 |
| 14. The person’s bra is visible (可以看見當事人的胸圍) | 0.80 | 1 | 0.83 | 1 | 1 | 1 | 0.83 | 1 | 1 | 1 | 0.83 | 1 |
| 15. Digitally altered images or videos that depict another person in a sexual way (such as those created using Photoshop or other editing software) (經過數碼修改的照片或影片，呈現當事人帶有性意味的形象  （例如使用 Photoshop 或其他編輯軟體所製作），包括移花接木等技術製作的虛假照片) | 0.80 | 1 | 0.83 | 1 | 0.88 | 1 | 0.67 | 1 | 1 | 1 | 0.67 | 1 |
| 16. Non-consensual sexual deepfakes (videos or images) created using deep learning artificial intelligence to replace, alter,  or mimic another person’s face or voice (使用深度學習人工智能技術，例如替換、更改或模仿當時人的臉部或聲音，  製作未經當事人同意的性深度偽造照片或影片) | 0.80 | 1 | 0.83 | 1 | 1 | 1 | 0.83 | 1 | 1 | 1 | 0.83 | 1 |
| **Average S-CVI** | **0.69** | **0.98** | **0.83** | **1** | **0.92** | **0.98** | **0.94** | **1** | **0.93** | **0.99** | **0.94** | **1** |

# Table S3. CVI of the items related to NIB-TFSA victimization.

| **Item** | **I-CVI** | | | | | | | | | | | |
| --- | --- | --- | --- | --- | --- | --- | --- | --- | --- | --- | --- | --- |
|  | **Relevance** | | | | **Appropriateness** | | | | **Clarity** | | | |
|  | **HM** | **BM** | **HF** | **BF** | **HM** | **BM** | **HF** | **BF** | **HM** | **BM** | **HF** | **BF** |
| 1. Received unwanted sexually explicit images or videos (收到不想要的色情或帶有性暗示的照片或影片) | 1 | 1 | 0.83 | 1 | 1 | 1 | 1 | 1 | 1 | 1 | 1 | 1 |
| 2. Received unwanted sexually explicit comments or texts (收到不想要的色情或帶有性暗示的評論或短訊) | 1 | 1 | 0.83 | 1 | 0.80 | 1 | 0.83 | 1 | 1 | 1 | 0.83 | 1 |
| 3. Received unwanted sexual requests (收到不想要的性請求) | 1 | 1 | 0.83 | 1 | 1 | 1 | 1 | 1 | 0.80 | 1 | 0.83 | 1 |
| 4. Being publicly posted online with offensive sexual comments about you (被人在網上公開發佈對您帶有性侮辱/性冒犯的評論) | 0.80 | 1 | 0.83 | 1 | 1 | 1 | 1 | 1 | 1 | 1 | 0.83 | 1 |
| 5. Being publicly posted online with personal details and/or pictures saying you are available to have sex (被人在網上公開發佈您的個人詳細資料和／  或照片，聲稱您可以提供性服務／可以和其他人發生性關係) | 0.80 | 1 | 0.83 | 1 | 1 | 1 | 1 | 1 | 1 | 1 | 1 | 1 |
| 6. Being publicly posted online with personal details and/or pictures saying someone wants to have sex with you (被人在網上公開發佈您的個人詳細資料和／  或照片，聲稱有人想和您發生性關係) | 0.80 | 1 | 0.67 | 1 | 1 | 1 | 0.83 | 1 | 1 | 1 | 0.83 | 1 |
| 7. Had an unwanted sexual experience with someone met online (與網上認識的人有不想要／不願意的性經歷) | 0.80 | 1 | 0.67 | 1 | 1 | 1 | 0.83 | 1 | 1 | 1 | 0.83 | 1 |
| 8. Received or being posted offensive and/or degrading messages, comments, or other content about your gender identity (收到或被張貼針對您性別認同的帶有冒犯性和／  或貶低、侮辱意味的訊息、評論或其他內容) | 0.80 | 1 | 0.67 | 1 | 1 | 1 | 0.67 | 1 | 1 | 1 | 0.67 | 1 |
| 9. Received or being posted offensive and/or degrading messages, comments, or other content about your sexual orientation (收到或被張貼針對您性傾向的帶有冒犯性和/或貶低、  侮辱意味的訊息、評論或其他內容) | 0.80 | 1 | 0.67 | 1 | 1 | 1 | 0.67 | 1 | 1 | 1 | 0.67 | 1 |
| 10. Received or being posted offensive and/or degrading messages, comments, or other content about your sex roles (收到或被張貼針對您性行為角色的帶有冒犯性和/或貶低、  侮辱意味的訊息、評論或其他內容) | 0.80 | 1 | 0.67 | 1 | 1 | 1 | 0.67 | 1 | 1 | 1 | 0.67 | 1 |
| 11. Received sexually violent threats, such as threats to rape you (收到性暴力的威脅，例如要強姦您) | 0.80 | 1 | 0.67 | 1 | 1 | 1 | 0.83 | 1 | 1 | 1 | 0.83 | 1 |
| 12. Described or visually represented unwanted sexual act against you (被人以言語、圖像或其他視覺方式描述對  您進行您不想要或不願意的性行為) | 0.80 | 1 | 0.83 | 1 | 1 | 1 | 0.83 | 1 | 1 | 1 | 1 | 1 |
| 13. Being pressured to engage in phone sex (被逼進行電話性愛) | 0.80 | 1 | 1 | 1 | 0.80 | 1 | 1 | 1 | 1 | 1 | 1 | 1 |
| 14. Being pressured to engage in sexual activity via chat room or video call (被逼通過聊天室或視像通話進行性行為) | 0.80 | 1 | 0.83 | 1 | 0.80 | 1 | 0.83 | 1 | 1 | 1 | 0.83 | 1 |
| 15. Being pressured to engage in sexual acts on a digital device (e.g., mobile phone, tablet or computer) (被逼在電子設備（例如手機、平板或電腦）上進行性行為) | 0.80 | 1 | 0.83 | 1 | 0.80 | 1 | 0.83 | 1 | 1 | 1 | 0.83 | 1 |
| 16. Being pressured to discuss sex-related topics on a digital device (e.g., mobile phone, tablet or computer) (被逼在電子設備（例如手機、平板或電腦）上討論性話題) | 0.80 | 1 | 0.83 | 1 | 0.80 | 1 | 1 | 1 | 0.80 | 1 | 1 | 1 |
| 17. Being pressured to send nude image(s) or video of myself on a digital device (e.g., mobile phone, tablet or computer) (被逼發送自己的裸體照片或影片) | 0.80 | 1 | 0.67 | 1 | 0.60 | 1 | 0.83 | 1 | 1 | 1 | 1 | 1 |
| 18. Being pressured to send sexually explicit messages on a digital device (e.g., mobile phone, tablet or computer) (被逼在電子設備（例如手機、平板或電腦）上發送含有性暗示的訊息) | 0.80 | 1 | 0.83 | 1 | 0.80 | 1 | 1 | 1 | 1 | 1 | 1 | 1 |
| 19. Your personal information and/or pictures were used without your consent to create a fake account for sexual purposes, such as arranging sexual hookups, sending sexual requests to others, and engaging in sexting. (您的個人資料和/或照片被盜用於開設假帳戶去從事與性有關的活動，  例如約別人進行性行為、向他人發送性請求和發送與性相關的短訊) | 0.80 | 1 | 0.83 | 1 | 1 | 1 | 1 | 1 | 1 | 1 | 1 | 1 |
| **Average S-CVI** | **0.83** | **1** | **0.78** | **1** | **0.92** | **1** | **0.88** | **1** | **0.98** | **1** | **0.88** | **1** |

# Table S4. CVI of the items related to NIB-TFSA perpetration.

| **Item** | **I-CVI** | | | | | | | | | | | |
| --- | --- | --- | --- | --- | --- | --- | --- | --- | --- | --- | --- | --- |
|  | **Relevance** | | | | **Appropriateness** | | | | **Clarity** | | | |
|  | **HM** | **BM** | **HF** | **BF** | **HM** | **BM** | **HF** | **BF** | **HM** | **BM** | **HF** | **BF** |
| 1. Sent unsolicited sexually explicit images or videos (未經當事人同意，擅自發送色情或帶有性暗示的照片或影片) | 0.80 | 1 | 0.67 | 1 | 0.80 | 1 | 0.83 | 1 | 1 | 1 | 0.83 | 1 |
| 2. Sent unsolicited sexually explicit comments or texts (未經當事人同意，擅自發送色情或帶有性暗示評論或短訊) | 0.80 | 1 | 0.83 | 1 | 0.60 | 1 | 1 | 1 | 0.80 | 1 | 1 | 1 |
| 3. Sent unsolicited sexual requests (未經當事人同意發出性請求) | 0.80 | 1 | 0.83 | 1 | 0.96 | 1 | 1 | 1 | 0.80 | 1 | 1 | 1 |
| 4. Publicly posted offensive sexual comments about others online (在網上公開發佈對別人帶有性侮辱／性冒犯的評論) | 0.80 | 1 | 0.83 | 1 | 1 | 1 | 1 | 1 | 1 | 1 | 1 | 1 |
| 5. Publicly posted personal details and/or pictures of a person online, indicating that the person is offering sex service or is available for sex (在網上公開發佈別人的個人資料和／或照片，  聲稱當事人可以提供性服務或可以與他人發生性關係) | 0.80 | 1 | 0.83 | 1 | 1 | 1 | 1 | 1 | 0.80 | 1 | 1 | 1 |
| 6. Publicly posted personal details and/or pictures of a person online, indicating that you/someone wants to have sex with that person (在網上公開發佈別人的個人資料和／或照片，  聲稱您或有人想和當事人發生性關係) | 0.80 | 1 | 0.67 | 1 | 1 | 1 | 1 | 1 | 1 | 1 | 1 | 1 |
| 7. Forced someone you met online to have sex with you (強逼您在網上認識的人與您發生性行為) | 0.80 | 1 | 0.83 | 1 | 0.60 | 1 | 0.83 | 1 | 1 | 1 | 0.83 | 1 |
| 8. Sent or posted offensive and/or degrading messages, comments, or other content about others’ gender identify (發送或張貼針對當事人性別認同的帶有冒犯性和／  或貶低、侮辱意味的訊息、評論或其他內容) | 0.80 | 1 | 0.83 | 1 | 1 | 1 | 1 | 1 | 1 | 1 | 1 | 1 |
| 9. Sent or posted offensive and/or degrading messages, comments, or other content about others’ sexual orientation (發送或張貼針對當事人性傾向的帶有冒犯性和／  或貶低、侮辱意味的訊息、評論或其他內容) | 0.80 | 1 | 0.83 | 1 | 1 | 1 | 1 | 1 | 1 | 1 | 1 | 1 |
| 10. Sent or posted offensive and/or degrading messages, comments, or other content about other’s sex roles (發送或張貼針對當事人性行為角色的帶有冒犯性和／  或貶低、侮辱意味的訊息、評論或其他內容) | 0.80 | 1 | 0.83 | 1 | 1 | 1 | 1 | 1 | 1 | 1 | 1 | 1 |
| 11. Sent sexually violent threats, such as threats to rape others (發送性暴力威脅，例如威脅要強姦別人) | 0.80 | 1 | 0.83 | 1 | 1 | 1 | 1 | 1 | 1 | 1 | 1 | 1 |
| 12. Described or visually represented an unwanted sexual act against others (以言語、圖像或其他視覺方式描述或呈現對當事人進行其不想要的性行為) | 0.80 | 1 | 0.83 | 1 | 1 | 1 | 1 | 1 | 1 | 1 | 1 | 1 |
| 13. Pressured others to engage in phone sex (強逼當事人進行電話性愛) | 0.80 | 1 | 0.83 | 1 | 0.80 | 1 | 0.83 | 1 | 1 | 1 | 0.83 | 1 |
| 14. Pressured others to engage in sexual activity via chat room or video call (強逼當事人通過聊天室或視像通話進行性行為) | 0.80 | 1 | 0.83 | 1 | 0.80 | 0.90 | 0.83 | 1 | 1 | 1 | 0.83 | 1 |
| 15. Pressured others to engage in sexual acts on a digital device (e.g., mobile phone, tablet or computer) (逼當事人在電子設備（例如手機、平板或電腦）上進行性行為) | 0.80 | 0.90 | 0.83 | 1 | 0.80 | 0.90 | 1 | 1 | 1 | 1 | 1 | 1 |
| 16. Pressured others to discuss sex- related topics on a digital device (e.g., mobile phone, tablet or computer) (強逼當事人在電子設備（例如手機、平板或電腦）上討論性話題) | 0.80 | 1 | 0.83 | 1 | 0.80 | 1 | 1 | 1 | 1 | 1 | 1 | 1 |
| 17. Pressured others to send nude image(s) or video of himself or herself on a digital device (e.g., mobile phone, tablet or computer) (強逼當事人發送自己的裸體照片或影片) | 0.80 | 1 | 0.83 | 1 | 0.80 | 1 | 1 | 1 | 1 | 1 | 1 | 1 |
| 18. Pressured others to send sexually explicit messages on a digital device (e.g., mobile phone, tablet or computer) (強逼當事人在電子設備（例如手機、平板或電腦）上發送有性暗示的訊息) | 0.80 | 1 | 0.83 | 1 | 0.80 | 1 | 1 | 1 | 1 | 1 | 1 | 1 |
| 19. Used others’ personal information and/or pictures without their consent to create a fake account for sexual purposes, such as arranging sexual hookups, sending sexual requests to others, and engaging in sexting (盜用別人的個人資料和／或照片，用於開設假帳戶去從事與性有關的活動，  例如約別人進行性行為、向他人發送請求和發送與性相關的短訊) | 0.80 | 1 | 0.83 | 1 | 1 | 1 | 1 | 1 | 1 | 1 | 1 | 1 |
| **Average S-CVI** | **0.80** | **0.99** | **0.81** | **1** | **0.88** | **0.99** | **0.96** | **1** | **0.97** | **1** | **0.96** | **1** |

# Table S5. CVI of the items related to OIPSV victimization.

| **Item** | **I-CVI** | | | | | | | | | | | |
| --- | --- | --- | --- | --- | --- | --- | --- | --- | --- | --- | --- | --- |
|  | **Relevance** | | | | **Appropriateness** | | | | **Clarity** | | | |
|  | **HM** | **BM** | **HF** | **BF** | **HM** | **BM** | **HF** | **BF** | **HM** | **BM** | **HF** | **BF** |
| 1. Insisting on having sex with you (but did not use physical force) (在沒有使用武力的情況下，對方堅持與您發生性行為) | 0.80 | 1 | 1 | 1 | 1 | 1 | 1 | 1 | 1 | 1 | 1 | 1 |
| 2. Using threats to force you to have sex (but did not use physical force) （在沒有使用武力的情況下，以威嚇來強迫對方與您發生性行為） | 0.80 | 1 | 1 | 1 | 1 | 1 | 1 | 1 | 1 | 1 | 0.83 | 1 |
| 3. Using physical force (such as hitting, holding down or using a weapon) to force you to have sex (以武力（例如打您、按住您、或使用武器）來強迫您與對方本人發生性行為) | 0.80 | 1 | 1 | 1 | 1 | 1 | 0.67 | 1 | 1 | 1 | 0.83 | 1 |
| 4. Insisting on having condomless sex with you (but did not use physical force) (在沒有使用武力的情況下，對方堅持要與您進行無套的性行為) | 0.80 | 1 | 1 | 1 | 0.80 | 1 | 1 | 1 | 1 | 1 | 1 | 1 |
| 5. Using threats to force you to have condomless sex (but did not use physical force) （與在網上認識的人見面時，您有沒有曾經在沒有使用武力的情況下，以威嚇來強迫對方與您發生無套的性行為） | 0.80 | 1 | 0.83 | 1 | 1 | 1 | 0.83 | 1 | 1 | 1 | 0.83 | 1 |
| 6. Using physical force (such as hitting, holding down or using a weapon) to force you to have condomless sex (以武力（例如打您、按住您、或使用武器）來逼您進行無套的性行為) | 0.80 | 1 | 1 | 1 | 1 | 1 | 1 | 1 | 1 | 1 | 1 | 1 |
| 7. Non-consensual condom removal during sexual activity, also known as 'stealthing' (性行為期間，在您不知情的情況下把安全套脫掉) | 0.60 | 1 | 1 | 1 | 1 | 1 | 1 | 1 | 1 | 1 | 1 | 1 |
| 8. Ejaculation in/on your body without your consent (沒有您的同意下，在您的身體内或表面射精) | 0.80 | 1 | 1 | 1 | 1 | 0.90 | 1 | 1 | 1 | 0.90 | 1 | 1 |
| 9. Intentionally transmitting HIV/ or other STI to you (故意將愛滋病／其他性病傳染給您) | 0.80 | 1 | 1 | 1 | 1 | 1 | 1 | 1 | 1 | 1 | 0.83 | 1 |
| **Average S-CVI** | **0.78** | **1** | **0.98** | **1** | **0.98** | **0.99** | **0.94** | **1** | **1** | **0.94** | **0.92** | **1** |

# Table S6. CVI of the items related to OIPSV perpetration.

| **Item** | **I-CVI** | | | | | | | | | | | |
| --- | --- | --- | --- | --- | --- | --- | --- | --- | --- | --- | --- | --- |
|  | **Relevance** | | | | **Appropriateness** | | | | **Clarity** | | | |
|  | **HM** | **BM** | **HF** | **BF** | **HM** | **BM** | **HF** | **BF** | **HM** | **BM** | **HF** | **BF** |
| 1. When meeting people online, have YOU ever insisted on having sex with others (but did not use physical force) (與在網上認識的人見面時，您有沒有曾經在沒有使用武力的情況下，堅持與對方發生性行為) | 0.60 | 1 | 0.83 | 1 | 0.80 | 1 | 0.83 | 1 | 1 | 1 | 0.83 | 1 |
| 2. When meeting people online, have YOU ever used threats to force others to have sex (but did not use physical force) (與在網上認識的人見面時， 您有沒有曾經在沒有使用武力的情況下，以威嚇來強迫對方與您發生性行為) | 0.60 | 1 | 0.67 | 1 | 0.80 | 1 | 0.50 | 1 | 1 | 1 | 0.50 | 1 |
| 3. When meeting people online, have YOU ever used physical force (such as hitting, holding down or using a weapon) to force others to have sex （與在網上認識的人見面時， 您有沒有曾經以武力（例如打對方、按住對方、或使用武器）來強迫對方與您發生性行為） | 0.60 | 1 | 0.67 | 1 | 0.80 | 1 | 0.67 | 1 | 1 | 1 | 0.67 | 1 |
| 4. When meeting people online, have YOU ever insisted on having condomless sex with others (but did not use physical force) （與在網上認識的人見面時，您有沒有曾經在沒有使用武力的情況下，堅持要與對方進行無套性行為） | 0.60 | 1 | 0.67 | 1 | 0.80 | 1 | 0.67 | 1 | 1 | 1 | 0.67 | 1 |
| 5. When meeting people online, have **YOU** ever used threats to force others to have condomless sex (but did not use physical force) （與在網上認識的人見面時，您有沒有曾經在沒有使用武力的情況下，以威嚇來強迫對方與您發生無套的性行為） | 0.60 | 1 | 0.67 | 1 | 0.80 | 1 | 0.67 | 1 | 1 | 1 | 0.67 | 1 |
| 6. When meeting people online, have **YOU** ever used physical force (such as hitting, holding down or using a weapon) to force others to have condomless sex （與在網上認識的人見面時，您有沒有曾經以武力（例如打對方、按住對方、或使用武器）來迫對方進行無套的性行為） | 0.60 | 1 | 0.83 | 1 | 0.80 | 1 | 0.83 | 1 | 1 | 1 | 0.83 | 1 |
| 7. When meeting people online, have **YOU** ever removed the condom during sexual activity without their consent (also known as 'stealthing') （與在網上認識的人見面時，您有沒有曾經在性行爲期間，在對方不知情的情況下把安全套脫掉） | 0.60 | 1 | 0.83 | 1 | 0.80 | 1 | 0.83 | 1 | 1 | 1 | 0.83 | 1 |
| 8. When meeting people online, have **YOU** ever ejaculated in/on other's body without their consent （與在網上認識的人見面時，您有沒有曾經在沒有對方的同意下，在對方的身體內或表面射精） | 0.60 | 1 | 0.83 | 1 | 0.80 | 1 | 0.83 | 1 | 1 | 1 | 0.83 | 1 |
| 9. When meeting people online, have **YOU** ever intentionally transmitted HIV or other STIs to others （與在網上認識的人見面時，您有沒有曾經故意將愛滋病病毒/其他性病傳染給對方） | 0.60 | 1 | 0.67 | 1 | 0.80 | 1 | 0.67 | 1 | 1 | 1 | 0.67 | 1 |
| **Average S-CVI** | **0.60** | **1** | **0.74** | **1** | **0.80** | **1** | **0.72** | **1** | **1** | **1** | **0.72** | **1** |
